# Preoperative Anxiolysis in Surgical Care Without Sedation or General Anesthesia: A Systematic Review

**DOI:** 10.3390/dj14060327

**Published:** 2026-06-01

**Authors:** Inesa Stonkutė, Dominykas Afanasjevas, Audra Janovskienė, Dainius Razukevičius, Žygimantas Petronis

**Affiliations:** 1 Faculty of Odontology, Medical Academy, Lithuanian University of Health Sciences, J. Lukšos-Daumanto 2, LT-50106 Kaunas, Lithuania; audra.janovskiene@lsmu.lt (A.J.); dainius.razukevicius@lsmu.lt (D.R.); zygimantas.petronis@lsmu.lt (Ž.P.); 2 Faculty of Medicine, Medical Academy, Lithuanian University of Health Sciences, A. Mickevičiaus g. 9, LT-44307 Kaunas, Lithuania; dominykas.afanasjevas@stud.lsmu.lt

**Keywords:** preoperative anxiety, oral surgery, local anesthesia, anxiolysis, benzodiazepines, pregabalin, melatonin, herbal medicine, dental anxiety

## Abstract

**Background/Objectives:** Preoperative anxiety is common in adult patients undergoing oral and dentoalveolar surgical procedures under local anesthesia and may impair cooperation, physiological stability, and overall treatment experience. While intravenous sedation and general anesthesia provide effective anxiolysis, they increase anesthetic exposure and recovery demands. Targeted preoperative anxiolysis offers a less invasive strategy to reduce anxiety while preserving responsiveness. However, approaches vary and standardized protocols are lacking. This systematic review evaluated the efficacy and safety of preoperative anxiolytic interventions—including both pharmacological and non-pharmacological strategies—in adult patients undergoing oral surgical procedures under local anesthesia without general anesthesia or deep sedation. **Methods**: The review adhered to the PRISMA 2020 guidelines and was prospectively registered in PROSPERO (CRD420261281592). Randomized and quasi-randomized controlled trials published between 2016 and 2026 were identified through structured searches of PubMed/MEDLINE, ScienceDirect, and Springer Nature Link. Eligible studies included adult patients undergoing oral surgery under local anesthesia and evaluated preoperative anxiolysis using validated instruments such as the Dental Anxiety Scale (DAS), State–Trait Anxiety Inventory (STAI), and Visual Analog Scale for Anxiety (VAS-A). Risk of bias was assessed using the Cochrane RoB 2 tool. Owing to methodological heterogeneity, results were synthesized narratively. **Results**: Eight trials (*n* = 617) met the inclusion criteria. Interventions included oral benzodiazepines, melatonin, pregabalin, herbal agents, nitrous oxide, and auriculotherapy. Benzodiazepines consistently reduced anxiety scores (*p* < 0.05) without significant interagent differences. Pregabalin at a dose of 150 mg significantly lowered STAI-S and VAS-A scores (*p* < 0.001). *Passiflora incarnata* was comparable to midazolam and superior to placebo, whereas *Erythrina mulungu* showed no effect. Melatonin results were inconsistent. Hemodynamics remained stable, and adverse events were mild. **Conclusions**: Preoperative anxiolysis under local anesthesia effectively reduces anticipatory anxiety in oral surgery, with benzodiazepines demonstrating the most consistent efficacy. Further standardized trials are warranted.

## 1. Introduction

Preoperative anxiety is a clinically significant psychological condition characterized by anticipatory fear and emotional distress associated with dental and surgical interventions, with oral and maxillofacial procedures consistently reported among those eliciting the highest anxiety levels [1]. In this context, anxiety is predominantly driven by anticipated pain, diminished perceived control, and the psychologically threatening characteristics of interventions performed under local anesthesia [2]. Elevated anxiety negatively affects patient behavior and compliance. It may lead to appointment avoidance, impaired cooperation, and reduced ability to process preoperative information, thereby complicating communication and perioperative management. Intraoperatively, anxious patients increase procedural difficulty and surgeon stress. This may impair technical performance and prolong operative time, ultimately compromising surgical conditions, patient comfort, and postoperative outcomes [3,4].

Despite widespread use, there is no consensus on optimal pharmacological strategies for preoperative anxiolysis in awake oral surgery [5]. This highlights the need for targeted anxiety-reduction strategies, particularly in awake patients undergoing oral surgical procedures. In oral and maxillofacial surgery, commonly implemented strategies include preoperative anxiolysis or minimal sedation to promote relaxation and facilitate patient cooperation while preserving protective reflexes and verbal responsiveness [6,7,8].

Preoperative anxiolysis comprises pharmacological or non-pharmacological interventions administered before surgery to attenuate anxiety and stress responses and is distinct from intraoperative conscious or deep sedation, which is primarily intended to facilitate operative conditions [6]. Its use is well established in oral and maxillofacial surgery, particularly in anxious or phobic patients, individuals with pronounced physiological stress responses, and those undergoing complex or prolonged procedures [9]. Clinical evidence indicates that anxiolytic interventions may improve tolerance of local anesthetic administration, stabilize cardiovascular parameters, enhance cooperation, and improve the overall treatment experience [8,10].

A broad range of pharmacological and non-pharmacological approaches have been described in the literature. Commonly investigated pharmacological agents include benzodiazepines, melatonin, gabapentinoids, inhalational nitrous oxide, and herbal preparations, administered at varying doses and at different preoperative intervals [11,12,13]. Non-pharmacological strategies such as music therapy and auriculotherapy have also been explored [14]. However, considerable heterogeneity exists regarding drug selection, dosing regimens, timing of administration, patient selection criteria, and anxiety assessment methods. This variability reflects the absence of standardized protocols or clear consensus guidelines for anxiolytic premedication management in oral surgery.

Given this diversity and the lack of an established therapeutic framework, a systematic evaluation of the available evidence is warranted. The aim of this systematic review is to critically evaluate and synthesize the available clinical evidence on both pharmacological and non-pharmacological anxiety-reduction strategies in adult patients undergoing oral and dentoalveolar surgical procedures under local anesthesia. Particular emphasis is placed on anxiolytic efficacy, physiological effects, safety outcomes, and methodological quality. By comparing evidence across different intervention types, this review seeks to support evidence-based clinical decision-making and to identify areas where further high-quality research is warranted.

## 2. Materials and Methods

### 2.1. Protocol and Registration

This systematic review was designed and reported in accordance with the Preferred Reporting Items for Systematic Reviews and Meta-Analyses (PRISMA) 2020 guidelines [15] (Supplementary File S1). To ensure transparency and reproducibility, the review protocol was registered prospectively in the PROSPERO database (registration number: CRD420261281592). The review question and eligibility criteria were defined a priori using the PICO framework (Population, Intervention, Comparator, Outcome), as presented in Table 1.

### 2.2. Eligibility Criteria

#### 2.2.1. Inclusion Criteria

Studies were included if they met all of the following criteria:

1. Randomized controlled or quasi-experimental clinical trials published in English between 2016 and 2026;
2. Enrolled adult patients undergoing oral surgical procedures;
3. Evaluated preoperative anxiety-reduction interventions, including pharmacological agents or non-pharmacological approaches;
4. Assessed anxiety using validated measurement instruments (e.g., STAI, DAS, MDAS, and VAS-A).

#### 2.2.2. Exclusion Criteria

Studies were excluded if they:

1. Primarily investigated general anesthesia, deep sedation, or intravenous sedation without a specific focus on preoperative anxiolysis;
2. Used observational, uncontrolled, or single-arm designs;
3. Involved non-oral surgical procedures;
4. Were case reports, narrative reviews, systematic reviews, editorials, or conference abstracts.

### 2.3. Information Sources and Search Strategy

A comprehensive electronic literature search was conducted to identify studies evaluating anxiolytic premedication in patients undergoing oral surgery. The search was performed between 3 February and 20 February 2026, using the following electronic databases: PubMed/MEDLINE, ScienceDirect, and Springer Nature Link.

The search strategy was developed in accordance with PRISMA guidelines and employed Boolean operators to combine key concepts related to anxiolytic interventions and oral surgical procedures. The primary search string applied across databases was (anxiolytics) AND (oral surgery). Search terms were adapted as needed for each database and included relevant synonyms and related terms.

The search was restricted to human studies published in the English language between January 2016 and January 2026. All records retrieved from the database searches were imported into EndNote, where duplicate entries were identified and removed prior to the screening process.

In addition, the reference lists of all included studies and relevant review articles were manually examined to identify any further eligible publications not captured by the electronic search.

### 2.4. Study Selection

Study selection was performed independently by three reviewers (I.S., D.A. and A.J.) according to predefined eligibility criteria. An initial screening of titles and abstracts was conducted to exclude clearly irrelevant records. Full-text articles were subsequently assessed for eligibility.

At each stage, studies were excluded based on predefined criteria, including: (1) focus on general anesthesia, deep sedation, or intravenous sedation without a specific emphasis on preoperative anxiolysis; (2) absence of validated anxiety outcome measures; (3) non-interventional study designs (e.g., observational studies, case reports, and reviews); (4) non-oral surgical procedures; and (5) insufficient reporting of intervention characteristics or outcomes.

Discrepancies were resolved through discussion among reviewers, with arbitration by a senior reviewer (Ž.P.) when necessary. The study selection process, including reasons for exclusion at the full-text stage, is summarized in the PRISMA flow diagram.

### 2.5. Data Extraction

Data were independently extracted by three reviewers (I.S., D.A. and A.J.) using a standardized data extraction form. Extracted data included:

- Author(s) and year of publication;
- Study design and setting;
- Sample size and participant characteristics;
- Type of oral surgical procedure;
- Description of anxiolytic intervention (type, dose, route, and timing);
- Comparator intervention;
- Anxiety assessment tools and timing of measurements;
- Physiological parameters related to anxiety;
- Reported adverse events.

Any discrepancies in data extraction were resolved by consensus.

Although validated anxiety instruments (e.g., DAS, STAI, and VAS-A) were employed across studies, standardized diagnostic thresholds distinguishing dental anxiety from dental phobia were not consistently applied. Consequently, analyses were conducted based on reported anxiety scores rather than formal diagnostic classifications. Baseline anxiety severity and anxiety-related eligibility criteria were additionally extracted and are summarized in Supplementary Table S1 [11,12,13,16,17,18,19,20].

### 2.6. Risk of Bias Assessment

The risk of bias of the included studies was assessed using the Revised Cochrane Risk of Bias tool for randomized trials (RoB 2) [21]. This tool was selected because all included studies employed randomized or quasi-randomized interventional designs and because RoB 2 is the recommended instrument for assessing internal validity in randomized controlled trials.

The RoB 2 tool evaluates potential sources of bias across five domains:

(1) Bias arising from the randomization process;
(2) Bias due to deviations from intended interventions;
(3) Bias due to missing outcome data;
(4) Bias in measurement of the outcome;
(5) Bias in selection of the reported result.

Each domain was assessed using the signaling questions and algorithmic guidance provided by the RoB 2 framework and was rated as “low risk of bias,” “some concerns,” or “high risk of bias.” An overall risk-of-bias judgment for each study was derived in accordance with Cochrane recommendations, based on the highest level of risk identified across the individual domains.

Risk of bias assessments were conducted independently by three reviewers (I.S., D.A. and A.J.). Any discrepancies between reviewers were resolved through discussion and consensus. When necessary, a fourth reviewer (Ž.P.) was consulted to arbitrate unresolved disagreements.

### 2.7. Data Synthesis

Due to substantial heterogeneity in the types of anxiolytic interventions, study designs, outcome measures, and timing of anxiety assessment, a qualitative narrative synthesis was undertaken. Included studies were summarized and compared descriptively, with findings grouped according to intervention category (pharmacological and non-pharmacological) and, where applicable, by pharmacological drug class.

A quantitative meta-analysis was not performed, as the clinical and methodological diversity of the included studies precluded meaningful statistical pooling. In particular, variability in anxiolytic agents, dosing regimens, comparator interventions, and anxiety assessment instruments limited the feasibility of calculating comparable effect estimates across studies.

## 3. Results

### 3.1. Study Selection

**Figure 1:**
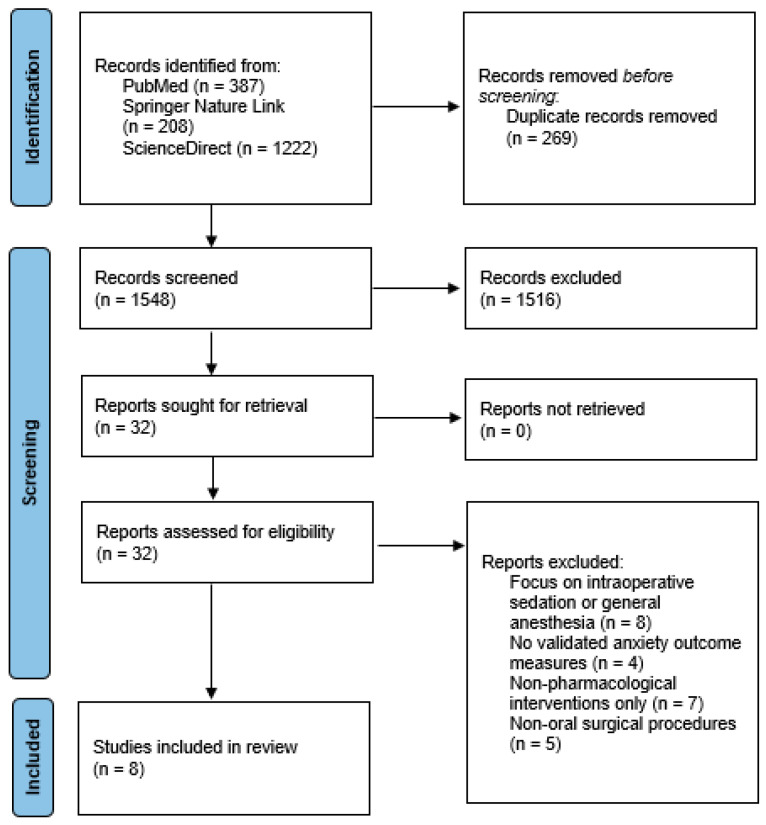
PRISMA flow diagram of study selection process.

The literature search identified 1817 records across the selected databases. After removing 269 duplicates, 1548 records were screened by title and abstract, of which 1516 were excluded due to irrelevance to the study focus. Thirty-two full-text articles were assessed for eligibility. Of these, 24 studies were excluded for the following reasons: focus on intraoperative sedation or general anesthesia (n = 8), absence of validated anxiety outcome measures (n = 4), evaluation of exclusively non-pharmacological interventions (n = 7), and inclusion of non-oral surgical procedures (n = 5). Ultimately, eight studies met the inclusion criteria and were included in the qualitative synthesis. The study selection process and detailed reasons for exclusion are presented in Figure 1.

### 3.2. Characteristics of Included Studies

A total of eight studies were included in the qualitative synthesis [11,12,13,16,17,18,19,20]. All were randomized or quasi-randomized clinical trials conducted in patients undergoing oral or dentoalveolar surgical procedures, most commonly mandibular third molar extraction. Additional procedures included upper third molar extraction and minor oral surgical interventions performed under local anesthesia.

Sample sizes across the included studies ranged from 30 to 200 participants. Most trials enrolled healthy adult patients (predominantly ASA I), with reported age ranges typically spanning 18 to 45 years. Where baseline anxiety was reported, levels were generally mild to moderate, although several studies specifically included patients with moderate to severe dental anxiety based on validated screening instruments. Across included trials, baseline anxiety severity varied. In the study by de Moares et al. [2], all participants (120/120) presented with moderate-to-severe anxiety (mean DAS 14.5 ± 2.2), corresponding to high dental anxiety thresholds. Mulla et al. [6] reported that 50% of participants (25/50) had high anxiety levels (DAS > 11), while Diniz et al. [4] exclusively included patients with elevated STAI scores (mean STAI-T 47.32 ± 7.29), indicative of high trait anxiety. In contrast, other trials did not restrict inclusion to high-anxiety individuals, and baseline scores suggested mixed or moderate anxiety distributions [1,3,5,7,8]. Phobia-level categorization (e.g., DAS ≥ 15) was not consistently reported. However, none of the included studies formally differentiated between dental anxiety and dental phobia based on standardized diagnostic criteria. Detailed baseline anxiety characteristics and phobia-level reporting across studies are presented in Supplementary Table S1.

Across studies, anxiety management was achieved primarily using pharmacological agents, including benzodiazepines, melatonin, gabapentinoids, herbal anxiolytics, and nitrous oxide. In selected trials, non-pharmacological interventions such as auriculotherapy were used as comparators. Anxiety was assessed using validated subjective instruments (e.g., Dental Anxiety Scale, Visual Analogue Scale for Anxiety, and State-Trait Anxiety Inventory) and, in several studies, complemented by physiological parameters such as heart rate, blood pressure and oxygen saturation. The key methodological and clinical characteristics of the included studies are summarized in Table 2.

### 3.3. Risk of Bias in Included Studies

**Figure 2:**
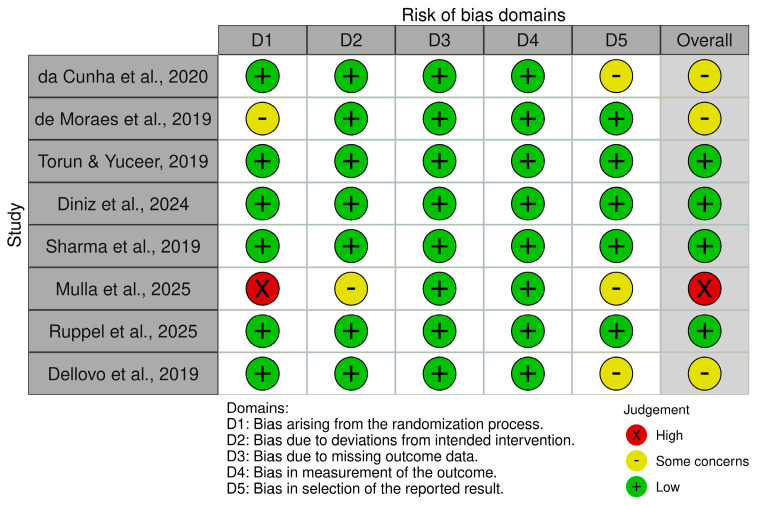
RoB 2 risk-of-bias traffic light plot showing domain-level judgments for each included study [11,12,13,16,17,18,19,20].

The risk of bias of the included studies was assessed using the Revised Cochrane Risk of Bias tool for randomized trials (RoB 2) [21], and the results are summarized in Table 3 and visually presented in Figure 2. Overall, most included studies were judged to be at low risk of bias across assessed domains. Two studies were rated as having some concerns, primarily related to selection of the reported result, while one quasi-experimental study was assessed as having a high risk of bias due to deviations from intended interventions and baseline group differences.

### 3.4. Results of Individual Studies

The effects of preoperative anxiolytic interventions on anxiety outcomes are summarized narratively below and schematically in Table 4, which presents a comparative overview of the findings by intervention class. Substantial heterogeneity was observed across studies in interventions, comparators, outcome measures, and timing of assessment.

#### 3.4.1. Benzodiazepines

Five studies including 450 participants evaluated oral benzodiazepines as anxiolytic therapy in oral surgical procedures under local anesthesia, predominantly mandibular third molar extraction (Table 3). Investigated agents included lorazepam, diazepam, midazolam, and alprazolam [11,12,17,18,20].

Across randomized trials, benzodiazepines were consistently associated with notable reductions in preoperative anxiety. In the trial by de Moares et al. [12], patients with moderate to severe anxiety showed a reduction in DAS scores from 14.5 ± 2.2 at baseline to approximately 11.5 postoperatively across midazolam, diazepam, and nitrous oxide groups (*p* < 0.05), without notable differences between agents (*p* > 0.05). Similarly, da Cunha et al. [11] reported that midazolam was superior to placebo (*p* < 0.0001) but did not differ from *Passiflora incarnata* (*p* = 0.79).

Comparative trials did not demonstrate clinically meaningful differences in anxiolytic efficacy between benzodiazepines. Sharma et al. [17] found comparable sedation and intraoperative cooperation with lorazepam and diazepam (*p* > 0.05), although lorazepam was associated with improved preoperative sleep (66% vs. 32%) and a higher incidence of retrograde amnesia (23 vs. 3 patients). In the quasi-experimental study by Mulla et al. [18], alprazolam administered to highly anxious patients (DAS 14.32 ± 2.04 vs. 4.68 ± 1.11; *p* = 0.001) resulted in substantial reductions in pulse rate and systolic blood pressure (*p* < 0.05), while oxygen saturation remained unchanged. Elevated preoperative pulse rate (OR 1.16; *p* = 0.017) and systolic blood pressure (OR 1.54; *p* = 0.002) were predictive of high anxiety.

In a randomized crossover trial by Dellovo et al. [20], anxiety reduction occurred in 86.7% of patients receiving midazolam and 83.3% receiving auriculotherapy (*p* = 0.74); however, midazolam was associated with notably greater retrograde amnesia and a higher frequency of adverse effects (*p* < 0.0001).

Overall, use of benzodiazepines was a reliable anxiety-reduction strategy across diverse study designs, with differences between agents primarily related to amnestic effects, sleep quality, and recovery characteristics rather than the magnitude of anxiety reduction.

#### 3.4.2. Melatonin

Two randomized controlled trials, including a total of 136 participants, evaluated the anxiolytic effects of melatonin administered preoperatively in patients undergoing mandibular third molar extraction under local anesthesia [13,19].

In the trial by Torun and Yuceer [13], melatonin was administered at a dose of 0.4 mg/kg orally 60 min preoperatively and compared with midazolam (0.2 mg/kg) and placebo. Baseline VAS anxiety scores were similar across groups (melatonin 7.40 ± 2.01; midazolam 7.27 ± 1.81; placebo 7.43 ± 1.59; *p* = 0.932). Post-medication anxiety scores were significantly lower overall (*p* < 0.001), with mean VAS values of 3.77 ± 3.13 in the melatonin group, 2.27 ± 1.19 in the midazolam group, and 5.23 ± 2.66 in the placebo group. Anxiety reduction was greater with midazolam than with melatonin (*p* < 0.05), although melatonin also produced a meaningful reduction compared with placebo (*p* = 0.016).

In contrast, Ruppel et al. [19] administered 15 mg of sublingual melatonin 45 min before surgery and did not observe meaningful differences between melatonin and placebo in intraoperative anxiety or discomfort outcomes (*p* > 0.05). Intraoperative pain scores were comparable (VAS 12.9 vs. 12.5; *p* = 0.67), and no measurable differences were observed in postoperative pain (*p* = 0.67), edema (*p* = 0.26), or trismus (*p* ≥ 0.50). Neither study demonstrated anxiolytic efficacy of melatonin comparable to benzodiazepines across all evaluated outcomes.

#### 3.4.3. Gabapentinoids

One randomized, triple-blind, split-mouth clinical trial evaluated pregabalin as preoperative anxiolytic premedication in patients undergoing bilateral impacted lower third molar extraction under local anesthesia (Table 3). Pregabalin (150 mg) was administered orally 1 h before surgery.

Baseline trait anxiety, assessed using the State-Trait Anxiety Inventory-Trait (STAI-T), indicated high anxiety levels (mean 47.32 ± 7.29). Compared with placebo, pregabalin was associated with a greater reduction in preoperative state anxiety, as measured by the State-Trait Anxiety Inventory-State (STAI-S) (41.19 ± 6.45 vs. 47.32 ± 6.47; *p* < 0.001) and the Visual Analogue Scale for Anxiety (VAS-A) (1.58 ± 0.81 vs. 2.61 ± 0.67; *p* < 0.001).

Pregabalin administration was accompanied by significant reductions in systolic and diastolic blood pressure and heart rate at several perioperative time points (*p* < 0.05), while oxygen saturation remained unchanged (*p* > 0.05). Sedation scores were higher in the pregabalin group (*p* < 0.01); however, patients remained cooperative and responsive throughout the procedures. Adverse effects were mild and infrequent [16].

#### 3.4.4. Herbal Anxiolytics

A randomized, triple-blind, placebo-controlled clinical trial, including 200 participants, investigated the preoperative anxiety-reduction effects of *Passiflora incarnata* (500 mg) and *Erythrina mulungu* (500 mg) administered orally 60 min prior to mandibular third molar extraction under local anesthesia (Table 3). The herbal interventions were compared with oral midazolam (15 mg) and placebo.

*Passiflora incarnata* exhibited anxiolytic effects comparable to midazolam (*p* = 0.79) and superior to placebo (*p* < 0.0001), as assessed using validated anxiety questionnaires and physiological parameters. In contrast, *Erythrina mulungu* did not demonstrate a statistically meaningful anxiolytic effect compared with placebo (*p* = 0.16). No statistically significant differences in physiological safety parameters were observed among groups (*p* > 0.05) [11].

#### 3.4.5. Nitrous Oxide and Non-Pharmacological Comparators

In the randomized clinical trial by de Moares et al. [12], nitrous oxide inhalation resulted in measurable reduction in anxiety scores compared with baseline (*p* < 0.05), with no statistically significant difference when compared with oral midazolam or diazepam (*p* > 0.05). Nitrous oxide was associated with reductions in anxiety as assessed using the Corah Dental Anxiety Scale; however, it was evaluated primarily as a comparative intervention rather than as a standalone preprocedural anxiolytic treatment.

In the crossover trial by Dellovo et al. [20], auriculotherapy meaningfully reduced anxiety relative to baseline (*p* < 0.05) and showed anxiolytic effects comparable to midazolam (*p* = 0.74). However, midazolam produced greater retrograde amnesia and a higher incidence of undesirable effects (*p* < 0.0001).

### 3.5. Synthesis of Results

Across the eight included studies, preoperative anxiolytic interventions resulted in variable effectiveness in reducing anxiety in patients undergoing oral and maxillofacial surgical procedures under local anesthesia [11,12,13,16,17,18,19,20]. Due to substantial heterogeneity in intervention types, study designs, anxiety assessment instruments, and timing of outcome measurement, results were synthesized narratively rather than quantitatively.

Pharmacological anxiolytics showed the most consistent anxiolytic effects. Benzodiazepines were associated with reliable reductions in preoperative anxiety across multiple randomized and quasi-experimental studies, regardless of the specific agent used [11,12,17,18,20]. No individual benzodiazepine was indicative of clear superiority in anxiolytic efficacy. Differences between agents were mainly confined to secondary outcomes, including sedation, amnesia, recovery profile, and adverse effects [11,12,17,20].

Evidence for non-benzodiazepine pharmacological agents was more limited. Pregabalin exhibited a marked reduction in preoperative anxiety compared with placebo in a single randomized trial, with additional benefits in hemodynamic stability and patient cooperation, without progression to procedural sedation [16]. In contrast, findings for melatonin were inconsistent, with one study reporting anxiolytic benefit compared with placebo and another showing no significant effect [13,19]. Where directly compared, melatonin did not achieve anxiolytic efficacy equivalent to benzodiazepines [13].

Herbal anxiolytics showed variable efficacy, with *Passiflora incarnata* demonstrating anxiolytic effects comparable to midazolam, whereas *Erythrina mulungu* did not differ from placebo [11].

Nitrous oxide and non-pharmacological interventions were primarily evaluated as comparators. Nitrous oxide revealed anxiolytic effects comparable to oral benzodiazepines but was not assessed as a standalone preoperative premedication [12]. Auriculotherapy produced reductions in anxiety similar to pharmacological anxiolytics in one study, though pharmacological agents generally achieved greater anxiety control and were associated with more pronounced sedative and amnestic effects [20].

Overall, the synthesis indicates that pharmacological anxiolysis—particularly with benzodiazepines—remains the most consistently effective approach for preoperative anxiety reduction in oral and maxillofacial surgical procedures without general anesthesia or deep sedation [11,12,17,18,20]. Alternative interventions identified anxiolytic potential in selected settings, offering anxiety reduction with fewer sedative and amnestic effects than benzodiazepines [11,13,16,19,20].

Reporting bias could not be formally assessed due to the limited number of studies included in each intervention category. The certainty of evidence was not formally assessed using the GRADE approach.

## 4. Discussion

Oral surgical interventions are required for a substantial proportion of patients in routine dental practice; however, a considerable number of individuals report clinically relevant anxiety, particularly in relation to procedures performed under local anesthesia [22].

An important methodological consideration concerns baseline anxiety severity in the included trials. Across studies, anxiety levels ranged from moderate to high, with some cohorts approaching commonly used phobia-level thresholds (e.g., DAS ≥ 15). However, formal diagnostic differentiation between dental anxiety and dental phobia was not consistently applied. As summarized in Supplementary Table S1, only a minority of trials explicitly recruited patients with moderate-to-high anxiety levels or reported phobia-level thresholds. Because clinical guidelines generally recommend adjunctive pharmacological anxiolysis primarily for patients with clinically relevant anxiety or phobia-level scores, the present findings should be interpreted within the context of the enrolled populations rather than generalized to all patients undergoing oral surgery under local anesthesia.

For patients with pronounced fear, oral surgery may be performed under intravenous sedation or general anesthesia. Nevertheless, many procedures can be safely completed under local anesthesia alone, and some patients are reluctant to undergo deeper sedation because of perceived risks, including respiratory depression, cardiovascular instability, postoperative nausea, and other systemic adverse effects described in the literature [23,24,25]. These considerations highlight the need for effective anxiety-reduction strategies that do not require escalation to intravenous sedation or general anesthesia.

When comparing outcome patterns across studies, an important distinction emerges between intravenous sedation-based protocols, general anesthesia, and targeted anxiety-reduction strategies under local anesthesia. Intravenous midazolam-based sedation has been shown to produce very high intraoperative comfort and overall satisfaction scores, frequently exceeding 90 on 100-point VAS-A scales, with strong amnestic effects [7,9]. Patient satisfaction in these studies correlated most strongly with intraoperative comfort (r ≈ 0.76, *p* < 0.01) and amnesia (r ≈ 0.57, *p* < 0.01), indicating that perceived procedural quality was largely driven by reduced awareness and limited recollection of the surgical experience [7]. However, preoperative reassurance scores were substantially lower, suggesting that anxiety reduction primarily occurred after sedative administration rather than before the procedure [7]. Moreover, in other intravenous sedation cohorts, a notable proportion of patients, exceeding 40%, still described the experience as at least somewhat uncomfortable despite adequate operative conditions [9].

Similar patterns are observed in studies evaluating general anesthesia for third molar surgery. In a retrospective comparison of intravenous sedation and general anesthesia, postoperative pain and overall satisfaction were high in both groups (96–100%), with no statistically significant difference in pain intensity between modalities (*p* > 0.05) [26]. However, general anesthesia was associated with substantially longer anesthesia duration and markedly prolonged recovery time compared with intravenous sedation [26]. These findings suggest that general anesthesia ensures unconsciousness and controlled operative conditions. However, it increases anesthetic exposure and recovery time without clear superiority in postoperative comfort. Furthermore, baseline anxiety tended to be higher among patients treated under general anesthesia, indicating that deeper anesthetic strategies may reflect psychological factors rather than purely procedural necessity [26].

Moreover, a clinical study comparing sedation and general anesthesia during third molar extraction reported that, although both modalities provided acceptable operative conditions, patients undergoing general anesthesia established a more negative psycho-emotional status on the day of surgery and experienced appreciably higher immediate postoperative pain scores (mean VAS 3.8 vs. 2.6; *p* < 0.05) compared with those treated under sedation. Importantly, baseline dental fear levels were comparable between groups, suggesting that deeper anesthetic strategies do not necessarily translate into superior psychological comfort outcomes [27].

In contrast, the findings of the present systematic review indicate that oral preoperative anxiolysis administered under local anesthesia markedly reduces anticipatory anxiety prior to surgical intervention, without inducing deep sedation or complete amnesia [11,12,13,16,17,18,19,20]. Across eight included trials, validated anxiety measures such as DAS, STAI, and VAS-A exhibited statistically significant reductions from baseline [12,17]. Importantly, these reductions were achieved while maintaining hemodynamic stability (*p* > 0.05 in most studies for heart rate, blood pressure, and oxygen saturation) and preserving full patient responsiveness. Adverse effects were generally mild and infrequent [11,12,13,16,17,18,19,20].

The findings of the present review are broadly consistent with previously published systematic reviews and meta-analyses evaluating anxiety-reduction strategies in dental and surgical settings. In particular, Steenen et al. (2024) [28] reported that pharmacological anxiolysis, especially benzodiazepines, demonstrates reliable efficacy in reducing dental anxiety, although variability exists depending on study design, patient characteristics, and outcome measures. Similarly, our analysis identified benzodiazepines as the most consistently effective intervention across included trials, without clear superiority among individual agents.

However, an important distinction of the present review is its specific focus on preoperative anxiolysis in oral surgical procedures performed under local anesthesia without deep sedation or general anesthesia. This more narrowly defined clinical context allows for clearer differentiation between anxiolytic and sedative effects, whereas prior reviews have often included broader sedation-based interventions, potentially confounding interpretation of anxiety-specific outcomes [28].

From a clinical perspective, these modalities represent a gradient of intervention intensity. General anesthesia maximizes unconsciousness but increases anesthetic exposure and recovery time [26]. Intravenous sedation provides high levels of comfort and amnesia with shorter recovery than general anesthesia but still requires monitoring and specialized infrastructure [7,9,26,29]. Targeted preoperative anxiolysis offers a less invasive alternative, achieving meaningful anxiety reduction while minimizing recovery burden and sedation-related complications [11,12,13,16,17,18,19,20].

Collectively, these findings support a graduated and individualized approach to anxiety management in oral surgery, reserving intravenous sedation or general anesthesia for selected high-anxiety or complex cases, while optimizing evidence-based preoperative anxiolytic strategies under local anesthesia whenever feasible.

## 5. Limitations

This systematic review has several limitations. First, the number of included studies was relatively small (n = 8), limiting the robustness and generalizability of conclusions. Second, substantial clinical and methodological heterogeneity was observed across studies in terms of anxiolytic agents, dosing regimens, timing of administration, outcome measures, and patient populations, which precluded quantitative meta-analysis. Third, most included studies involved relatively small sample sizes and predominantly healthy adult populations (ASA I), limiting applicability to medically complex patients. Fourth, non-pharmacological interventions were underrepresented, restricting comparative evaluation across intervention types. An additional limitation is the inability to differentiate between dental anxiety and dental phobia, as included studies relied on validated anxiety scales without consistent use of diagnostic thresholds or psychiatric classification criteria. Finally, publication bias could not be formally assessed due to the limited number of studies per intervention category.

## 6. Conclusions

This systematic review suggests that pharmacological pre-surgical anxiolytic therapy under local anesthesia can reduce anticipatory anxiety in selected adult patients undergoing oral surgery. However, the strength of this conclusion is limited by heterogeneity across studies, variable baseline anxiety severity, and inconsistent reporting of phobia-level classification. Among the evaluated interventions, oral benzodiazepines showed the most consistent anxiolytic efficacy across randomized and quasi-experimental trials, without clear superiority between individual agents. Pregabalin and *Passiflora incarnata* yielded anxiolytic potential in single high-quality trials, whereas evidence for melatonin was inconsistent.

Importantly, anxiety reduction was achieved while maintaining hemodynamic stability and full patient responsiveness, with generally mild and infrequent adverse effects. In contrast to intravenous sedation and general anesthesia, which primarily enhance comfort through sedation depth and amnesia, preoperative anxiolysis appears to reduce anxiety through psychological stabilization without increasing anesthetic exposure or recovery burden.

These findings support a graduated, individualized approach to anxiety management in oral surgery. For many patients, targeted preoperative anxiety modulation under local anesthesia may provide clinically meaningful anxiety reduction while avoiding the risks, monitoring requirements, and prolonged recovery associated with intravenous sedation or general anesthesia. Further high-quality randomized trials with standardized outcome measures are warranted to strengthen the evidence base and facilitate development of clinical guidelines. Preoperative anxiolysis should therefore be considered primarily for patients presenting with clinically relevant anxiety or phobia-level scores, rather than as a routine adjunct for all oral surgical procedures under local anesthesia.

## Figures and Tables

**Table 1:** PICO framework.

Component	Description
Population (P)	Adult patients undergoing oral or dentoalveolar surgical procedures, including third molar extraction, dental implant placement, and other minor oral surgical interventions
Intervention (I)	Pharmacological anxiolytic agents administered preoperatively
Comparator (C)	Placebo, no intervention, standard care, alternative pharmacological anxiolytics, or non-pharmacological anxiety-reduction interventions
Outcome (O)	Preoperative anxiety reduction assessed using validated instruments * (STAI, DAS, MDAS, and VAS-A), with secondary outcomes including physiological indicators of anxiety (heart rate, blood pressure, and oxygen saturation) and reported adverse events

* STAI—State-Trait Anxiety Inventory; DAS—Dental Anxiety Scale; MDAS—Modified Dental Anxiety Scale; VAS-A—Visual Analogue Scale for Anxiety.

**Table 2:** Key characteristics of included studies.

Author (Year)	Study Design	Number of Patients	Surgical Procedure	Intervention	Comparator	Anxiety Measure(s)	Timing of Assessment
da Cunha et al., 2020 [11]	Randomized, triple-blind, placebo-controlled parallel RCT	200	MTME	*Passiflora incarnata* 500 mg or *Erythrina mulungu* 500 mg (60 min preoperatively)	Midazolam 15 mg; placebo	Anxiety questionnaires; heart rate; blood pressure; SpO_2_	Preoperative; intraoperative
de Moares et al., 2019 [12]	Randomized clinical trial	120	MTME	Midazolam 7.5 mg (oral)	Diazepam (oral); nitrous oxide inhalation	Corah Dental Anxiety Scale (DAS)	Preoperative; postoperative
Torun & Yuceer, 2019 [13]	Randomized, double-blind clinical trial	90	Impacted MTME	Melatonin 0.4 mg/kg (60 min preoperatively)	Midazolam 0.2 mg/kg; placebo	Visual Analog Scale for Anxiety (VAS-A); cognitive and psychomotor tests	Baseline; 60 min preoperatively
Diniz et al. (2024) [16]	Randomized, triple-blind, split-mouth RCT	31	Bilateral impacted MTME	Pregabalin + dexamethasone (administered 1 h preoperatively)	Placebo + dexamethasone	STAI-S; STAI-T; VAS-A	Baseline; preoperative; intraoperative; postoperative
Sharma et al., 2019 [17]	Double-blind, crossover, prospective, and randomized study	50	Bilateral MTME	Lorazepam 2.5 mg (night before surgery + 1 h preoperatively)	Diazepam 10 mg (night before surgery + 1 h preoperatively)	Ramsay Sedation Scale; recall of visual stimuli; Pittsburgh Sleep Quality Index	Night before surgery + 1 h preoperatively
Mulla et al., 2025 [18]	Quasi-experimental controlled study	50	Minor oral surgical procedures	Alprazolam 0.25 mg (30 min preoperatively)	No anxiolytic intervention	Physiological parameters (pulse rate, blood pressure, respiratory rate, SpO_2_)	Preoperative; intraoperative; postoperative
Ruppel et al., 2025 [19]	Randomized, double-blind, placebo-controlled RCT	46	MTME	Melatonin 15 mg sublingual (45 min preoperatively)	Placebo	Intraoperative discomfort questionnaire; VAS	Preoperative; intraoperative; postoperative
Dellovo et al., 2019 [20]	Randomized, double-blind, crossover RCT	30	Bilateral MTME	Oral midazolam 15 mg + sham auriculotherapy	Auriculotherapy + placebo tablet	Anxiety questionnaires; blood pressure; heart rate; SpO_2_	Baseline; day of surgery; follow-up

MTME—mandibular third molar extraction.

**Table 3:** Risk of bias assessment of included studies using the Cochrane RoB 2 tool.

Study	Randomization Process	Deviations from Intended Interventions	Missing Outcome Data	Measurement of Outcome	Selection of Reported Result	Overall Risk of Bias
da Cunha et al., 2020 [11]	Low	Low	Low	Low	Some concerns	Some concerns
de Moares et al., 2019 [12]	Some concerns	Low	Low	Low	Low	Some concerns
Torun & Yuceer, 2019 [13]	Low	Low	Low	Low	Low	Low
Diniz et al., 2024 [16]	Low	Low	Low	Low	Low	Low
Sharma et al., 2019 [17]	Low	Low	Low	Low	Low	Low
Mulla et al., 2025 [18]	High	Some concerns	Low	Low	Some concerns	High
Ruppel et al., 2025 [19]	Low	Low	Low	Low	Low	Low
Dellovo et al., 2019 [20]	Low	Low	Low	Low	Some concerns	Some concerns

**Table 4:** Summary of anxiolytic effects by intervention type.

Intervention Type	Included Studies (n)	Surgical Procedures	Anxiety Measures Used	Summary of Anxiolytic Effects
Benzodiazepines (lorazepam, diazepam, midazolam, alprazolam)	5	Mandibular and maxillary third molar extraction, minor oral surgery	DAS ^1^; VAS-A ^2^; sedation scales; physiological parameters	Preoperative benzodiazepine administration was consistently associated with reductions in anxiety-related outcomes compared with baseline or control conditions across randomized and quasi-experimental studies. No clear superiority among individual benzodiazepines was observed.
Melatonin	2	Mandibular third molar extraction	VAS-A ^2^; intraoperative discomfort measures	Evidence for anxiolytic efficacy was mixed. One study reported reduced anxiety compared with placebo, while another found no clinically meaningful difference. Anxiolytic effects were generally inferior to midazolam when directly compared.
Gabapentinoids (pregabalin)	1	Bilateral impacted lower third molar extraction	STAI-S ^3^; STAI-T ^4^; VAS-A ^2^	Pregabalin administered preoperatively was associated with a notable reduction in preoperative state anxiety compared with placebo when coadministered with dexamethasone
Herbal anxiolytics (*Passiflora incarnata*, *Erythrina mulungu*)	1	Mandibular third molar extraction	Anxiety questionnaires; physiological parameters	*Passiflora incarnata* indicated anxiolytic effects comparable to midazolam, whereas *Erythrina mulungu* did not differ meaningfully from placebo.
Nitrous oxide (comparator)	1	Third molar extraction	Corah Dental Anxiety Scale	Nitrous oxide showed similar anxiolytic efficacy to oral benzodiazepines but was evaluated primarily as a comparator rather than a standalone preoperative anxiolytic intervention.
Non-pharmacological interventions (auriculotherapy)	1	Mandibular third molar extraction	Anxiety questionnaires, physiological parameters	Auriculotherapy demonstrated anxiety-reducing effects comparable to midazolam, with fewer reported adverse effects.

^1^ DAS—Dental Anxiety Scale; ^2^ VAS-A—Visual Analogue Scale for Anxiety; ^3^ STAI-S (State Anxiety)—assesses transient, situation-specific anxiety; ^4^ STAI-T (Trait Anxiety)—assesses stable anxiety predisposition.

## Data Availability

The original contributions presented in this study are included in the article/Supplementary Material. Further inquiries can be directed to the corresponding author.

## Supplementary Materials

The following supporting information can be downloaded at: https://www.mdpi.com/article/10.3390/dj14060327/s1, File S1: PRISMA 2020 checklist; Table S1: Baseline anxiety severity and anxiety-related eligibility criteria across included studies.
