# Supplementary material for: Preoperative Anxiolysis in Surgical Care Without Sedation or General Anesthesia: A Systematic Review
Source: Dent J (Basel). 2026 Jun 1;14(6):327. doi: 10.3390/dj14060327 (PMC13298367; doi:10.3390/dj14060327)
Supplement: Supplementary file 1 [file dentistry-14-00327-s001.zip › dentistry-4198224-Table S1.pdf]

Supplementary Table S1. Baseline anxiety severity and anxiety-related eligibility criteria across included studies

Study	Anxiety instrument	Baseline mean ± SD	Anxiety inclusion criteria	Phobia-level threshold reported	Number of high-anxiety patients
da Cunha et al. 2020 [1]	Anxiety questionnaires	NR	No predefined anxiety-based eligibility criteria	NR	NR
de Moraes et al. 2019 [2]	DAS	NR; moderate-severe anxiety required	Moderate-severe anxiety (DAS-based inclusion)	NR	120 (all participants)
Torun & Yuceer 2019 [3]	VAS-A	NR	No anxiety-based eligibility criteria	NR	NR
Diniz et al. 2024 [4]	STAI-T; STAI-S; VAS-A	STAI-T 47.32 ± 7.29	High or very high anxiety required (STAI-based)	Yes (STAI-based classification applied)	31 (all participants)
Sharma et al. 2019 [5]	Ramsay Sedation Scale (no formal anxiety scale)	NR	No anxiety-based eligibility criteria	NR	NR
Mulla et al. 2025 [6]	DAS	NR; stratified by DAS score	Anxious (DAS >11) vs non-anxious (DAS <10)	Yes (DAS-based grouping applied)	25 anxious patients
Ruppel et al. 2025 [7]	VAS	NR	No anxiety-based eligibility criteria	NR	NR
Dellovo et al. 2019 [8]	Anxiety questionnaires	NR	No predefined anxiety-based eligibility criteria	NR	NR

1. da Cunha, R.S.; Amorim, K.S.; Gercina, A.C.; de Oliveira, A.C.A.; Menezes, L.d.S.; Groppo, F.C.; Souza, L.M.A. Herbal medicines as anxiolytics prior to third molar surgical extraction. A randomized controlled clinical trial. *Clin. Oral Investig.* **2020**, *25*, 1579–1586. https://doi.org/10.1007/s00784-020-03468-1.
2. de Moares, M.B.; Barbier, W.S.; Raldi, F.V.; Nascimento, R.D.; dos Santos, L.M.; Sato, F.R.L. Comparison of Three Anxiety Management Protocols for Extraction of Third Molars With the Use of Midazolam, Diazepam, and Nitrous Oxide: A Randomized Clinical Trial. *J. Oral Maxillofac. Surg.* **2019**, *77*, 2258.e1–2258.e8. https://doi.org/10.1016/j.joms.2019.06.001.
3. Torun, A.C.; Yüceer, E. Should Melatonin Be Used as an Alternative Sedative and Anxiolytic Agent in Mandibular Third Molar Surgery? *J. Oral Maxillofac. Surg.* **2019**, *77*, 1790–1795. https://doi.org/10.1016/j.joms.2019.02.045.

4. Diniz, J.A.; Dourado, A.C.A.G.; Barbirato, D.d.S.; de Oliveira, M.S.V.; Lira, V.L.B.d.O.d.; Filho, S.M.C.d.M.; da Silveira, K.G.; Filho, J.R.L. Evaluation of the effects of pregabalin and dexamethasone coadministration on preemptive multimodal analgesia and anxiety in third molar surgeries: A triple-blind randomized clinical trial. *Clin. Oral Investig.* **2024**, *28*, 304. https://doi.org/10.1007/s00784-024-05700-8.
5. Sharma, V.; Singh, A.; Sharma, P.; Kaur, S.; Zutshi, A. Comparative Study Between Oral Lorazepam and Diazepam as Sedation in Oral and Maxillofacial Surgery. *J. Maxillofac. Oral Surg.* **2018**, *18*, 256–259. https://doi.org/10.1007/s12663-018-1096-1.
6. Mulla, M.F.; Pagare, V.; Changule, G.R.; Doiphode, A.; Mulla, M.; Jawarker, R.; Kaul, D.; Gupta, S. Evaluating the Anxiolytic Efficacy of Oral Alprazolam in Modulating Vital Parameters During Minor Oral Surgical Procedures: A Quasi-experimental Study. *Cureus* **2025**, *17*, e77651. https://doi.org/10.7759/cureus.77651.
7. Ruppel, C.; Rosa, H.H.; Cardoso, R.B.; Rigo, N.M.; Soto, V.C.; Bortoluzzi, M.C. Preoperative 15 mg of melatonin for surgical discomfort, pain, edema and trismus in mandibular third molar surgery: A randomized double-blind placebo-controlled clinical trial. *Clin. Oral Investig.* **2025**, *29*, 571. https://doi.org/10.1007/s00784-025-06649-y.
8. Dellovo, A.; Souza, L.; de Oliveira, J.; Amorim, K.; Groppo, F. Effects of auriculotherapy and midazolam for anxiety control in patients submitted to third molar extraction. *Int. J. Oral Maxillofac. Surg.* **2019**, *48*, 669–674. https://doi.org/10.1016/j.ijom.2018.10.014.
